# The changing picture of object substitution masking: reply to Di Lollo (2014)

**DOI:** 10.3389/fpsyg.2014.01004

**Published:** 2014-09-09

**Authors:** Endel Põder

**Affiliations:** Department of Experimental Psychology, Institute of Psychology, University of TartuTartu, Estonia

**Keywords:** visual masking, object substitution, attention, modeling, reentrant, feed-forward

In his recent comment, Di Lollo (2014) criticizes my proposal (Põder, 2013) that the attentional gating model (Reeves and Sperling, 1986; Sperling and Weichselgartner, 1995) might be the most simple and reasonable explanation for the results of object substitution masking (OSM) experiments. He argues that OSM cannot be explained without a reentrant hypotheses-testing mechanism (as proposed in Di Lollo et al., 2000). A closer look at his arguments reveals that they are partly based on an inaccurate interpretation of my study, and partly, on some highly problematic assumptions about visual processing.

The goal of my study (Põder, 2013) was to understand the mechanisms behind the results of a typical OSM experiment with varied duration of masker and set-size. There were two main points in my study that are relevant in the present context. First, I analyzed the computational model (CMOS) proposed by Di Lollo et al. (2000) and found it to be identical with the attentional gating model, which has no direct relationship with any kind of “reentrant processing.” Second, I proposed an improved mechanism of attention to be combined with this model (or other possible masking models). I have never proposed or tested any new model of masking.

Di Lollo (2014) seems to have a rather subjective view of my study. He criticizes something named Põder's feed-forward account (or model) of OSM, which is supposedly based on two assumptions: reduction of signal to noise ratio (SNR) as a result of integrated noise from the masker, and delayed deployment of attention. Both assumptions are declared to be wrong or at least unjustified.

As I mentioned, I have not built a new model of OSM but just reinterpreted Di Lollo et al.'s (2000) CMOS. Therefore, these assumptions can only be the assumptions underlying CMOS, and actually, they are. I believe that CMOS was a reasonably good model for OSM experiments and that its main assumptions cannot be fundamentally wrong. However, Di Lollo (2014) missed some possibly important details. In CMOS, SNR was reduced not only because of accumulating “noise” from the masker but also because of decay of the target signal. Neither Di Lollo et al. (2000) nor Põder (2013) supposed that SNR is proportional (or inversely proportional) to the duration of the masker.

The model that was tested in my study tried to explain the set-size effects better than the simple attention deployment mechanism used in CMOS. My model assumes that the set-size effect is caused by an initial stage of divided attention. In this model masking *per se* is independent of set-size. A similar idea about the invariance of masking to set-size was independently discovered by Argyropoulos et al. (2013). My model with divided attention goes a bit further and proposes a plausible explanation for the observed set size effects.

Having explained away the set-size effect, a simple masking effect remains. In my study (Põder, 2013), I did not attempt to reveal its exact mechanisms. I indicated that the combination of the decay of the target signal, integration of the masker signal, and a delayed attention as implemented in CMOS (Di Lollo et al., 2000) might do the job. However, there are many other possibilities. After the removal of the burden to explain set-size effects, the classic models of masking (Weisstein, 1968; Bridgeman, 1971; Francis, 1997) that were analyzed in Francis and Hermens (2002) become fully applicable for OSM (note that the unsatisfactory modeling of attention/set-size effects was the main problem with these models; Di Lollo et al., 2002). Of course, Di Lollo's reentrant hypotheses-testing idea can be included in the candidate list too, as well as Bachmann's (1994) non-specific amplification idea. Hopefully, future studies will be able to discriminate between these models.

Although the set-size effect has been quite convincingly separated from the masking effect, some role of attention in OSM is still not excluded. In a recent study, Pilling et al. (2014) found a modest effect of spatial pre-cueing in one out of the five experiments. Up to now, nobody has explained away the position uncertainty and pre-cueing effects reported in earlier studies (Enns and Di Lollo, 1997; Neill et al., 2002; Tata and Giaschi, 2004; Luiga and Bachmann, 2007). If attention is still important then the models of masking developed by Smith et al. (Smith and Wolfgang, 2004; Smith et al., 2009) or by Bridgeman (2007) may be considered.

Di Lollo (2014) mainly argues against a CMOS-like masking account, but apparently supposes that any kind of essentially feed-forward model cannot explain masking with a sparse trailing masker. The majority of his arguments are based on a quite strange view on the visual system. Di Lollo (2014) seems to ignore the hierarchical nature of visual processing and assume that all the masking-related processes should occur at some low-level retinotopic layer. Therefore, only retinotopic picture-level noise, integration, and masking are possible. The real visual system consists of at least 4–5 (possibly more) processing levels (e.g., DeYoe and van Essen, 1988; Riesenhuber and Poggio, 1999). The target and masker signals are processed and temporally and spatially integrated throughout this hierarchy. Thus, the “noise” from irrelevant stimuli may interact with relevant signals at any level of processing. The higher levels are increasingly invariant to spatial positions and combine all visual features including motion. It is therefore not surprising at all that the dots far from the target, or a masker that was retinotopically moved away from the target location (Lleras and Moore, 2003), can still interact at higher object recognition levels. Note that the higher-level masking does not need anything like sending perceptual hypotheses back to the lower levels.

A large part of Di Lollo's (2014) critique is directed against using SNR in the models of OSM (although the idea itself was introduced by Di Lollo et al., 2000). Di Lollo (2014) argues that a noisy representation of visual objects is not consistent with the phenomenal experience of not seeing them as noisy pictures; and with clearly different effects of the external pixel noise compared to four-dot masker. His arguments apparently challenge some points of traditional psychophysics. In usual psychophysical models (e.g., Macmillan and Creelman, 2005), noise is a random trial-by-trial variability of internal representations that causes incorrect perceptual decisions. This noise can make a letter A look like a letter B, or like a chicken, or like a blank screen, in some trials. We can manipulate this noise (or SNR) by varying stimulus contrast, size, or exposure duration, presenting distractors, or forward or backward maskers, pre-cueing attention, simultaneous eye movements, etc., besides adding external pixel noise. There is no reason to suppose that the decision-level noise should be visible and look like noise within a single image.

New studies have forced Di Lollo and colleagues to make some changes to their theory. The original account of OSM (Di Lollo et al., 2000) was heavily based on both reentrant hypotheses testing and deployment of attention. The Argyropoulos et al. (2013) results indicated that something is wrong with this theory. The simplest way out was to leave out attention. However, attention had a key role in CMOS and in the predictions related to the Di Lollo et al. (2000) theory. Jannati et al. (2013) found an innovative solution. Nominally, they removed attention but attributed its properties to “reentrant processing.” In the original model (Di Lollo et al., 2000), the reentrant processing was supposed to be perpetual generation and testing perceptual hypotheses with periodicity of about 13 ms. In their new account (Jannati et al., 2013), the reentrance “arrives” at about 80–120 ms after stimulus onset, a typical delay of focusing spatial attention (e.g., Cheal and Lyon, 1991). Overall, their revised theory still follows the attentional gating logic of CMOS. At the same time, they claim that their experiment falsifies the attentional gating account of OSM. A closer look at their arguments reveals that their description of the “attentional gating model” does not contain attention at all. It is not surprising that such a model cannot fit any (old or new) experimental results.

In conclusion, I would describe the present situation as follows. The attentional gating idea effectively explained the effects of attention and simplified the problem of OSM tremendously. Now, one may take a single target stimulus with a common-onset masker and present them at a fixed position of the visual field, with full attention available, and try to observe OSM. There is a chance that Di Lollo (or somebody else) can demonstrate the action of reentrant hypotheses-testing mechanism in that simple experiment. It would be an interesting and surprising (at least for me) finding. But it would not contradict my attentional gating account of OSM experiments with set-size variation.

## Conflict of interest statement

The author declares that the research was conducted in the absence of any commercial or financial relationships that could be construed as a potential conflict of interest.
